# IMPLEMENTING A VIRTUAL, AGE-FRIENDLY INTERGENERATIONAL PROGRAM

**DOI:** 10.1093/geroni/igac059.1411

**Published:** 2022-12-20

**Authors:** Tina Newsham, Elizabeth Fugate-Whitlock, Natalie Quinn, Sky Bergman

**Affiliations:** UNC Wilmington, Wilmington, North Carolina, United States; University of North Carolina Wilmington, Wilmington, North Carolina, United States; University of North Carolina Wilmington, Wilmington, North Carolina, United States; Cal Poly, San Luis Obispo, San Luis Obispo, California, United States

## Abstract

As the University of North Carolina Wilmington (UNCW) seeks to become more age-friendly and stakeholders pursue endorsement of the Age-Friendly University principles, a virtual intergenerational mentoring project was implemented. The aim was to connect the community’s older adults, including retired faculty, with current students. Older adults and students were paired and asked to engage in a series of conversations, watch a documentary, and attend a Q&A with the filmmaker. The goals were to improve social connectedness and well-being, improve expectations regarding aging for members of both groups, and to decrease ageism. Impacts of the project were assessed through the Expectations Regarding Aging scale prior to any interactions between partners and again at the end of the project along with an open-ended questionnaire about their experiences. Expectations regarding aging improved on 75% of the items, and participants indicated qualitatively that their experiences with the intergenerational interactions were positive.

